# Aged Garlic Extract Improves Adiponectin Levels in Subjects with Metabolic Syndrome: A Double-Blind, Placebo-Controlled, Randomized, Crossover Study

**DOI:** 10.1155/2013/285795

**Published:** 2013-02-28

**Authors:** Diego Gómez-Arbeláez, Vicente Lahera, Pilar Oubiña, Maria Valero-Muñoz, Natalia de las Heras, Yudy Rodríguez, Ronald Gerardo García, Paul Anthony Camacho, Patricio López-Jaramillo

**Affiliations:** ^1^Dirección de Investigaciones, Fundación Oftalmológica de Santander (FOSCAL), Torre Milton Salazar, Primer Piso, Calle 155A No. 23-09, El Bosque, Floridablanca, Santander, Colombia; ^2^Instituto de Investigaciones, Escuela de Medicina, Universidad de Santander (UDES), Bucaramanga, Colombia; ^3^Departamento de Fisiología, Facultad de Medicina, Universidad Complutense de Madrid, Spain

## Abstract

*Background*. Garlic (*Allium sativum*) has been shown to have important benefits in individuals at high cardiovascular risk. The aim of the present study was to evaluate the effects of the administration of aged garlic extract (AGE) on the risk factors that constitute the cluster of metabolic syndrome (MS). *Methods and Design*. Double-blind, crossover, randomized, placebo-controlled clinical trial to assess the effect of 1.2 g/day of AGE (Kyolic), for 24 weeks of treatment (12 weeks of AGE and 12 weeks of placebo), on subjects with MS. *Results*. The administration of AGE increased the plasma levels of adiponectin (*P* = 0.027). No serious side effects associated with the intervention were reported. *Conclusion*. The present results have shown for the first time that the administration of AGE for 12 weeks increased plasma adiponectin levels in patients with MS. This suggests that AGE might be a useful, novel, nonpharmacological therapeutic intervention to increase adiponectin and to prevent cardiovascular (CV) complications in individuals with MS.

## 1. Introduction

Metabolic syndrome (MS) is characterized by the presence of insulin resistance, low-degree inflammation, dysglycemia, low plasma high-density lipoprotein cholesterol (HDL-C), increased triglycerides (TG), elevated blood pressure, and abdominal obesity [1]. MS has been associated with an increased risk of type 2 diabetes mellitus (DM2) and cardiovascular diseases (CVDs) [1, 2]. The prevalence of MS varies between 15% and 40%, being greater in the population of Hispanic origin [3].

Abdominal obesity is considered a key characteristic of MS, which is related to decreased insulin-mediated glucose uptake [4]. Adipose tissue is known to express and secrete a variety of adipokines, including leptin, adiponectin, resistin, and visfatin, as well as cytokines and chemokines such as tumor necrosis factor-alpha (TNF-*α*), interleukin-6 (IL-6), and monocyte chemoattractant protein-1 (MCP-1) [5–8]. The release of adipokines by either adipocytes or adipose tissue-infiltrated macrophages plays a key role in the development of insulin resistance and DM2, as well as the increased risk of cardiovascular disease associated with obesity. Renin-angiotensin system components are also activated in adipose tissue, leading to hypertension and insulin resistance [4]. Adiponectin is considered to be a protective protein with antidiabetic, anti-inflammatory, and antiatherogenic effects [9]. Reduced plasma adiponectin levels have been reported in obese individuals, particularly in those with visceral obesity, and have been negatively correlated with insulin resistance. Furthermore, decreased adiponectin levels were found to be associated with a higher incidence of DM2 [4]. Leptin was shown to promote the development of atherosclerosis by inducing oxidative stress in endothelial cells, increasing platelet aggregation, and hypertrophy and proliferation of vascular smooth muscle cells [4]. Additionally, it was shown that a high leptin level predicts subsequent development of DM2 [6]. Thus, leptin/adiponectin imbalance has a key role in the metabolic alterations associated with obesity [5–10].

Multiple therapeutic approaches such as renin-angiotensin system blockers and inhibitors, statins as well as nutrient and dietary interventions [11–14], have been proposed to reduce metabolic and cardiovascular risk in patients with MS. Garlic (*Allium sativum *L.) has been used as a nutrient with beneficial cardiovascular effects [15]. However, the beneficial effects of garlic are offset by the fact that fresh garlic causes indigestion and that its pungent odor lingers on breath and skin [16]. An alternative source of garlic that is odorless and rich in antioxidants is aged garlic extract (AGE) [17]. AGE has shown beneficial effects in several alterations related to the development of cardiovascular diseases, such as antioxidant and antithrombotic properties [18–20]. Thus, in the present study we aimed to investigate the effects of AGE on adipokines, inflammatory substances, endothelial function, and metabolic risk factors that constitute the cluster of metabolic syndrome in an urban Colombian population.

## 2. Materials and Methods

### 2.1. Study Design

Double-blind, crossover, randomized, placebo-controlled clinical trial to assess the effect of AGE (Kyolic) on the cardiovascular risk factors of subjects with MS.

### 2.2. Population

Men and women over 18 years old with diagnosis of MS, attending primary health care clinics from the metropolitan area of Bucaramanga, Colombia. The MS diagnosis was based on the presence of central obesity (waist circumference ≥90 cm (male), ≥80 cm (female)) and two of the following criteria: TG ≥150 mg/dL, HDL-C <40 mg/dL (male), <50 mg/dL (female), blood pressure ≥130/85 mmHg, and fasting plasma glucose ≥100 mg/dL. The exclusion criteria were (1) allergies to garlic; (2) current treatment with lipid-lowering drugs, antihypertensive drugs, and/or hypoglycemic medications; (3) psychiatric disorders that prevent proper decision making; (4) patients with infections or inflammatory assets; (5) presence of coronary artery disease, with a current or past ischemic event; (6) presence of severe chronic or terminal illnesses; and (7) presence of diseases that compromise the immune system.

### 2.3. Procedures

**Figure 1:**
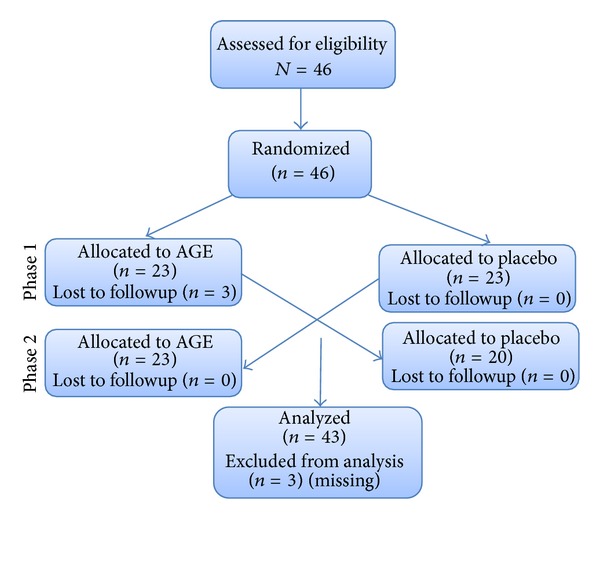
CONSORT diagram showing the flow of participants through each phase of the randomized crossover trial.

This study was registered in ClinicalTrials.gov with the identifier code NCT01168700. The study was approved by the ethical committee of the Cardiovascular Foundation in Bucaramanga, Colombia. All subjects provided written informed consent before entering the study. Patients were randomly assigned by blocks to receive either 1.2 g/day of AGE (Kyolic) or placebo, and after 12 weeks of supplementation, the treatment was invested for another 12 weeks (Figure 1). Each treatment was provided in an identical capsule that was taken twice daily with breakfast and dinner (2 capsules of each). All subjects received routine recommendations of lifestyle changes (having a diet lower in fat and sugar and increasing physical activity with 30 minutes/day of moderate walking). Participants were followed up every four weeks with clinical evaluations and registration of potential undesirable effects and use of any other medication. During the baseline and at the end of each phase of treatment (week 12 and week 24), the following were determined.

#### 2.3.1. Anthropometrical Measurements

Weight, height, body mass index (BMI), waist (WC) and hip circumferences (HC), and blood pressure.

#### 2.3.2. Biochemical Determinations

Routine clinical tests were processed in the Clinical Research Laboratory from the Ophthalmological Foundation of Santander-FOSCAL, Floridablanca, Colombia. Measurements of adipokines and inflammatory factors were performed at the Department of Physiology, Faculty of Medicine, Complutense University of Madrid, Spain. Glycemia and lipid profile were quantified by using a routine colorimetric method (Biosystem BTS-303 Photometric, Barcelona, Spain). Interleukin-6, adiponectin, and C-reactive protein were measured using an immunoassay (R&D Systems, MN, USA).

#### 2.3.3. Endothelial Function

Endothelial function was evaluated by flow mediated vasodilatation (FMV). The FMV was performed using a high-resolution Doppler ultrasound, measuring the changes in diameter of the brachial artery in response to increased blood flow (reactive hyperemia). This method was previously standardized in our population [21, 22].

#### 2.3.4. Statistical Analysis

The averages and proportions obtained in a descriptive analysis for all clinically relevant variables measured during the baseline evaluation were compared. Then, the treatment effect of the crossover design was evaluated through the difference in change between baseline versus posttreatment according to the intervention phase. Based on the frequencies distribution of the outcome variables, the Student's *t*-test or Wilcoxon signed-ranks test was used. In outcome variables where significant differences were observed, further analysis of changes was performed using analysis of covariance (ANCOVA), adjusting by phase, treatment, and their interaction (treatment × phase) to determine if changes were due to a carryover effect. All analyses were conducted using Stata statistical software, release 11.0 (Stata Corporation, College Station, TX, USA). A *P* < 0.05 was considered statistically significant.

## 3. Results

The 46 patients included in the study were distributed in two sequences of treatment: AGE-placebo and placebo-AGE. Three subjects (all of them of the first group) voluntarily discontinued the treatment during the phase 1. Demographic, anthropometric, and biochemical characteristics obtained in the 43 participants who completed the study are shown in Table 1. A significant difference in age was found between the AGE-placebo and the placebo-AGE groups at the baseline.

At the end of the study the crossover analysis was conducted, and a significant difference in the adiponectin delta was found comparing AGE versus Placebo, Δ: 313.79 (95%IC: −48.34~675.92) versus Δ: −271.88 (95%IC: −649.64~105.87), respectively (Table 2). The ANCOVA confirmed that the significant difference in adiponectin was due to the treatment, not the phase of the study (no carryover effect) as no significant changes were observed in the interaction treatment phase (Table 3). No significant changes were observed in any of the other anthropometrical measurements, endothelial function, and biochemical variables (Table 2). No serious side effects were associated with AGE administration.

## 4. Discussion

The present study demonstrates for the first time that the administration of AGE to subjects with MS for 12 weeks increased adiponectin plasma concentrations. The ANCOVA indicated that this outcome was not due to a carryover effect.

Our group previously reported that in dyslipidemic subjects, the presence of coronary artery disease is associated with an elevation of certain inflammatory markers but not with further endothelial dysfunction [23]. In the present study, after the AGE intervention there were no significant changes either in endothelial function or in inflammation, which may relate both to the short period of intervention and the participation of subjects with low cardiovascular risk. However, there was a significant increase in adiponectin, an anti-inflammatory adipokine with cardioprotective properties [24].

Low adiponectin levels are observed in obese subjects with and without severe coronary atherosclerosis and in subjects with abdominal obesity [10, 25], and decreased adiponectin levels (<4 *μ*g/mL) are associated with a twofold increase in the prevalence of coronary heart disease, independent of other cardiovascular risk factors [26]. Moreover, hypoadiponectinemia is associated with insulin resistance and DM2 [27, 28], as well as atherosclerosis and hypertension [29].

Adiponectin exerts an anti-inflammatory effect through activation of its three receptors (AdipoR1, AdipoR2, and T-cadherin) [9]. The activation of AdipoR1 and R2 results in increased hepatic and skeletal muscle fatty acid oxidation, increased skeletal muscle lactate production, reduced hepatic gluconeogenesis, increased cellular glucose uptake, and inhibition of inflammation and oxidative stress [30]. Activation of T-cadherin is protective in vascular endothelial cells against oxidative stress-induced apoptosis [31]. Several mechanisms have been suggested to explain the anti-inflammatory effects of adiponectin, including direct actions on inflammatory cells, actions on NF-*κ*B, and interaction with TNF-*α* [9]. It has been demonstrated that adiponectin inhibits the expression of adhesion molecules in endothelial cells and inhibits smooth muscle cell proliferation, the differentiation of monocytes into macrophages, as well as the formation of foam cells and the secretion of TNF-*α* by macrophages [32–34]. Also, increased adiponectin levels are related to improvement in the differentiation of preadipocytes into adipocytes, which is usually impaired in obese subjects [35]. In fact, 1,2-vinyldithiin (1,2-DT), a garlic-derived organosulfur compound, has been shown to affect the differentiation of human preadipocytes into adipocytes [36]. Interestingly, a significant reduction of the expression of the two major adipogenic transcription factors, PPAR*γ*2 and CCAAT/enhancer binding protein (C/EBP*α*), was observed in 1,2-DT-treated preadipocytes. The 1,2-DT-mediated decrease in PPAR*γ*2 expression is associated with reduced PPAR*γ* activity, suggesting that the negative effect of 1,2-DT on preadipocytes differentiation could be mainly due to an inhibitory effect on PPAR*γ*2, the master regulator of adipogenesis. The role of these mechanisms of action of 1,2-DT in the beneficial effects of AGE increasing the levels of adiponectin remains to be elucidated. Additionally, our results showing that a short period of AGE administration increases the adiponectin level suggest that the effect of AGE improving the insulin resistance could be another new interesting mechanism to explain the well-known beneficial cardiometabolic effect of garlic.

Another mechanism that could be associated with the adiponectin increase is the nitric oxide (NO) pathway. There appears to be a reciprocal relationship between adiponectin and NO [37]. Adiponectin increases the stability of eNOS mRNA and half-life, enhances the association of eNOS with Hsp90, and stimulates the phosphorylation of eNOS, which together lead to increased NO production [38, 39]. Moreover, NO appears to positively regulate adiponectin levels [40]. It has been suggested that AGE could increase NO bioavailability [40, 41] by (a) increasing cellular antioxidant capacity by providing cellular thiol antioxidants like cysteine and reduced glutathione, (b) maintaining functionally relevant levels of tetrahydrobiopterin and preventing oxidative inactivation of tetrahydrobiopterin, which prevents NO synthase uncoupling and superoxide anion generation, and (c) maintaining NO bioavailability in endothelial cells even under conditions of increased vascular oxidant stress [41]. AGE is rich in water-soluble organosulfur bioactive compounds such as S-allylcysteine and S-allylmercaptocysteine which are cellular donors of thiol containing reducing equivalents [41] and as such might explain the cardiovascular benefits of AGE.

In summary, we showed for the first time that AGE administration for 12 weeks increases adiponectin levels. The importance of this observation in the prevention of CVD remains to be determined, and further and larger studies are needed.

## Figures and Tables

**Table 1:** Baseline demographic, anthropometrical, endothelial function and biochemical characteristics of the general population.

Variable	Global	AGE-placebo Group (*n* = 20)	Placebo-AGE group (*n* = 23)	*P* value
Age (years)	40.79 (10.71)	44.75 (10.5)	37.34 (9.8)	0.02^∗,Ψ^
SBP (mm Hg)	123.73 (15.63)	127 (15)	120 (15)	0.15*
DBP (mm Hg)	81.59 (10.86)	82 (12)	80 (9)	0.56*
BMI (kg/m^2^)	33.07 (5.01)	33.7 (5.4)	32.5 (4.5)	0.54*
WC (cm)	100.44 (10.32)	102.4 (11.6)	98.7 (8.8)	0.23*
HC (cm)	109.42 (8.89)	108.8 (8.4)	109.8 (9.4)	0.46*
Glucose (mg/dL)	87.12 (14.99)	91.85 (17.53)	83 (11.19)	0.10*
Cholesterol (mg/dL)	198.61 (30.82)	201.9 (36.61)	195.73 (25.24)	0.51*
LDL (mg/dL)	116.96 (28.16)	119.25 (35.10)	114.95 (20.98)	0.61*
HDL (mg/dL)	37.47 (5.68)	37.51 (6.55)	37.42 (4.94)	0.87*
TAG (mg/dL)	220.91 (71.96)	225.65 (80.07)	216.78 (65.65)	0.88^#^
CRP (mg/L)	5.79 (4.73)	7.04 (5.73)	4.70 (3.39)	0.21*
IL-6 (units/mL)	1.97 (0.88)	1.93 (0.87)	2.00 (0.90)	0.68*
Adiponectin (ng/mL)	5936.56 (1813.74)	5516.7 (1665.24)	6301.65 (1893.59)	0.15*
Leptin (ng/mL)	26.19 (19.28)	27.29 (18.64)	25.22 (20.17)	0.60^#^
Insulin (*μ*U/mL)	22.26 (25.87)	28.81 (36.07)	16.58 (9.03)	0.10^#^
HOMA index	5.04 (6.51)	6.82 (9.03)	3.49 (2.22)	0.05^#^
FMV (%)	11.06 (5.98)	10.68 (5.39)	11.38 (6.37)	0.70*

SBP: systolic blood pressure; DBP: diastolic blood pressure; BMI: body mass index; WC: waist circumference; HC: hip circumference, LDL-C: low density lipoprotein cholesterol; HDL-C: high density lipoprotein cholesterol; TG: triglycerides; CRP: C-reactive protein; IL-6: interleukin-6; FMV: flow-mediated vasodilatation. Values are expressed as mean ± SD. *AGE group versus placebo group with independent Student's *t*-test. ^#^AGE group versus placebo group with Wilcoxon signed-ranks test. ^Ψ^ *P* < 0.05.

**Table 2:** Change differences in the anthropometrical measurements, endothelial function, and biochemical characteristics in the crossover analysis.

Parameters	AGE	Placebo	*P* value
	Change differences (*n* = 43)	Change differences (*n* = 43)	
SBP (mm Hg)	−2.59 ± 1.91	−1.72 ± 1.60	0.727*
DBP (mm Hg)	−1.07 ± 1.32	−0.31 ± 1.17	0.670*
BMI (kg/m^2^)	0.01 ± 0.21	−0.11 ± 0.17	0.952^#^
WC (cm)	−0.99 ± 0.47	0.32 ± 0.50	0.062*
HC (cm)	−0.91 ± 0.39	−0.39 ± 0.58	0.462^#^
WHR	−0.001 ± 0.004	−0.005 ± 0.005	0.358*
Glucose (mg/dL)	2.04 ± 1.68	3.46 ± 1.87	0.766^#^
Cholesterol (mg/dL)	−4.41 ± 4.47	6.0 ± 4.27	0.172^#^
LDL-C (mg/dL)	3.94 ± 5.32	6.51 ± 4.46	0.869^#^
HDL-C (mg/dL)	−1.64 ± 0.86	−1.16 ± 0.69	0.911^#^
TG (mg/dL)	−18.76 ± 12.42	−3.98 ± 13.84	0.453^#^
CRP (mg/L)	0.21 ± 0.85	0.07 ± 0.77	0.976^#^
IL-6 (units/mL)	0.08 ± 0.16	0.01 ± 0.18	0.682 ^#^
Adiponectin (ng/mL)	313.79 ± 179.44	−271.88 ± 187.18	0.027^∗Ψ^
Leptin (ng/mL)	−1.67 ± 1.68	−0.79 ± 1.26	0.993^#^
Insulin (*μ*U/mL)	−2.94 ± 2.60	2.26 ± 1.38	0.269^#^
HOMA index	−0.67 ± 0.70	0.67 ± 0.34	0.142^#^
FMV (%)	−0.81 ± 5.09	−1.34 ± 9.78	0.836*

SBP: systolic blood pressure; DBP: diastolic blood pressure; BMI: body mass index; WC: waist circumference; HC: hip circumference; WHR: waist-hip ratio; LDL-C: low density lipoprotein cholesterol; HDL-C: high density lipoprotein cholesterol; TG: triglycerides; CRP: C-reactive protein; IL-6: interleukin 6; FMV: flow-mediated vasodilatation. Values are expressed as mean ± SEM. *AGE group versus placebo group with Student's *t*-test. ^#^AGE group versus placebo group with Wilcoxon signed-ranks test. ^Ψ^ *P* < 0.05.

**Table 3:** Analysis of covariance (ANCOVA), adjusting by phase, treatment, and their interaction (treatment × phase).

Parameter	AGE	Placebo	*P* value	ANCOVA	
	Change differences (*n* = 43)	Change differences (*n* = 43)		*P* value treatment	*P* value phase
Adiponectin (ng/mL)	313.79 ± 179.44	−271.88 ± 187.18	0.027^∗Ψ^	0.031^&^	0.428

^Ψ^P < 0.05.  ^&^ *P* < 0.05 ANCOVA model. *P* value treatment and phase: analysis of covariance.
